# Point-of-Care Ultrasound Education and Use in Conflict Settings: Lessons From the War in Ukraine

**DOI:** 10.7759/cureus.108252

**Published:** 2026-05-04

**Authors:** Stanislav Kravchuk, Yurii Mashika, Matthew Hapij, Tanya Bucierka, Andrew Liteplo

**Affiliations:** 1 Gastroenterology, Danylo Halytsky Lviv National Medical University, Lviv, UKR; 2 Anesthesia and Critical Care, Lviv Military Medical Clinical Hospital of Border State of Ukraine, Lviv, UKR; 3 Emergency Medicine, Massachusetts General Hospital, Boston, USA; 4 Emergency Medicine, MedGlobal, Eugene, USA

**Keywords:** barriers, battlefield, education, novice, point-of-care ultrasound, trauma, trauma imaging, war

## Abstract

Introduction

Point-of-care ultrasound (POCUS) has a well-established role in acute medical care and is particularly valuable in conflict zones where access to advanced imaging is limited. To aid Ukrainian healthcare providers, POCUS courses were implemented, and doctors received education and ultrasound probes to use on the frontlines. The objectives of this study were to evaluate the use of POCUS by novice users in conflict-related medical environments, using the war in Ukraine as a case study. We sought to identify how and where medical personnel use ultrasound after an introductory POCUS course, and what barriers to use exist.

Methods

This was a cross-sectional survey of former participants in two-day POCUS training courses organized by Razom for Ukraine and the Christian Medical Association of Ukraine between January 2023 and September 2024. Applications taught included Focused Assessment with Sonography for Trauma (FAST), cardiac, lung, deep vein thrombosis (DVT), soft tissue, renal, hepatobiliary, soft tissue, nerve blocks, vascular access, musculoskeletal, and ocular. All course participants were medical personnel managing soldiers injured in battle. A link to the survey was distributed by Telegram, a messaging platform. Participation was voluntary and anonymous. This research was deemed to be exempt by the Institutional Review Board (IRB).

Results

The survey was sent to 352 participants. A total of 40% (140/352) of participants responded, of which 87% (122/140) were doctors. Participants worked in stabilization points (56%, 78/140), in military hospitals (31%, 43/140), and in medical transport (14%, 19/140). A total of 64% (89/140) used POCUS at least once a day. The most commonly used applications were FAST (89%, 124/140), pneumothorax (72%, 101/140), vascular access (62%, 87/140), soft tissue (54%, 75/140), and nerve blocks (44%, 62/140). The most common educational barriers to use were image interpretation (47%, 66/140) and image acquisition (31%, 44/140). The most common technological problems were damage to the probe or cord (36%, 50/140), a short battery lifespan (32%, 45/140), and not having access to a machine (26%, 36/140).

Conclusions

After a brief training, novice users regularly used POCUS in the care of wounded soldiers in Ukraine. FAST, pneumothorax, vascular access, soft tissue, and nerve blocks were the most frequently used applications. Future efforts should focus on additional education on image interpretation and acquisition, and on procurement of more ultrasound machines.

## Introduction

Point-of-care ultrasound (POCUS) has a well-established role in acute medical care. A long-required component of all emergency medicine training, it is now a ubiquitous tool used by almost all specialties [1]. Its value comes from being able to make real-time diagnoses of life-threatening conditions in a rapid and safe manner directly at the bedside. POCUS differs from traditional ultrasound in that the medical provider caring for the patient acquires images, interprets them, and immediately integrates the findings into medical decision-making. POCUS benefits patients in almost all settings where it is used, but these benefits are even more pronounced in resource-limited areas, austere environments, in underdeveloped countries, and in times of war [2-4].

Armed conflicts have devastating effects on healthcare infrastructure, and the war in Ukraine represents a contemporary example of these challenges [5]. Hospitals in medical facilities have been intentionally targeted and destroyed [6]. Doctors and other healthcare providers have taken on new roles within the medical system outside of their areas of training and gone to work at stabilization points and field hospitals on the battlefield to care for acutely wounded soldiers. Resources in these centers are often limited, including medical imaging. POCUS has been shown to have many uses in wartime settings [7-8]. Since the escalation of the war in Ukraine in 2022, numerous charitable organizations and individuals have purchased and donated ultrasound machines to help Ukraine’s medical system care for its injured defenders. The miniaturization of ultrasound technology over the past decade has made it possible for this technology to be exceedingly portable and thus much more versatile than other imaging modalities such as radiographs, computerized tomography, and magnetic resonance imaging [9].

The major challenge of effectively using POCUS is that a provider needs training and education. As POCUS was not in wide use prior to the escalation of war in 2022, training courses have been designed by experts and taught to novice medical personnel [10]. At the conclusion of these courses, participants were given an ultrasound probe to use on the frontlines. The objectives of this study were to evaluate the use of POCUS by these novice users inside and outside of the traditional hospital settings during the war in Ukraine. We sought to identify which POCUS applications are most commonly used by medical personnel after an introductory POCUS course, and what barriers to use exist. Such information may inform the design of future ultrasound education programs for medical personnel working in conflict zones, humanitarian crises, and other austere environments.

## Materials and methods

Study design

This was a cross-sectional study investigating the use of and barriers to POCUS by medical personnel in the war in Ukraine. The goal of the study was to obtain information about real use of POCUS in war after a brief training course, in order to best inform future educational and practical efforts to bring meaningful POCUS use to the frontlines. A 41-question survey was developed through literature review and expert consensus by a group of emergency physicians from the United States and Ukraine with expertise in POCUS. The survey was piloted for content and response process validity. Survey questions assessed demographics of providers, locations of use, and frequency of use. Additionally, details about application-specific use were asked, as were barriers to use. The voluntary, anonymous survey was determined to be exempt from review by the IRB (RedCap #1774).

Study setting and population

Survey respondents were participants of previous POCUS educational trainings that had been organized and sponsored by three organizations: Razom for Ukraine, a 501(c)(3) U.S. organization; the Christian Medical Association of Ukraine, a nonprofit charitable organization based in Lviv, Ukraine; and MedGlobal, a humanitarian charitable nongovernmental organization based in Chicago, IL, USA. Courses took place between January 2023 and September 2024.

Participants had various medical backgrounds (doctors, nurses, medical assistants, and medical volunteers) and all managed wounded soldiers during the war in Ukraine. Participants were trained by POCUS instructors from the abovementioned humanitarian and medical organizations. Typical courses included 14 hours of education and consisted of online and in-person training. The basic POCUS course involved three hours of online education via six video-recorded lectures and subsequent testing. The aim was to ensure an understanding of the principles of working with ultrasound. Subsequent in-person instruction took place over two days and consisted of short in-person lectures intertwined with longer hands-on practice during which participants practiced POCUS under the supervision of a trainer. Courses typically consisted of 15 participants, three instructors, and 3-4 ultrasound machines.

POCUS courses taught numerous applications of ultrasound including extended Focused Assessment with Sonography in Trauma (FAST) (abdominal fluid, pneumothorax, hemothorax, pericardial effusion), lung ultrasound (B-lines, consolidation), deep vein thrombosis ultrasound (DVT), abdomen (hepatobiliary, kidney, urinary bladder, appendix), ocular ultrasound (optic nerve sheath diameter, eye injury), musculoskeletal (tendon rupture, bone fracture), soft tissue (foreign bodies, abscess), cardiac (left ventricular function, right ventricular strain), vascular access and regional anesthesia.

At the conclusion of the training, many participants were given a personal Butterfly IQ+ ultrasound probe (Butterfly Network, Inc., Burlington, MA, USA). Hundreds had been purchased by Razom for Ukraine, as were the tablets to which these probes connected [11]. Participants underwent ultrasound training at various times (1-20 months) prior to the study survey. They were in various stages of deployment.

Sample size and sampling method

This was an exploratory, cross-sectional survey study, and no formal a priori sample size calculation was performed. We aimed to survey all individuals who had participated in POCUS training courses organized by the sponsoring organizations during the study period. A convenience sampling approach was used, with recruitment conducted via direct messaging to eligible participants through existing communication channels. All participants who received the survey link and chose to respond were included in the analysis.

Study protocol

The survey (Appendix 1) was distributed via Telegram Messenger (Dubai, United Arab Emirates), a popular and secure cloud-based mobile and desktop messaging app. Participants were already in contact with course trainers via this platform prior to initiation of the study. Participants received a direct message from a POCUS course instructor with a link to a survey designed in SurveyMonkey (Dublin, Ireland), a secure digital platform designed to distribute surveys. The survey was distributed in both Ukrainian and English. Participants were asked single-choice, multiple-choice, and open-ended questions.

Data analysis

Descriptive statistics were used to characterize demographic information, location of use, frequency of use, application-specific data, barriers to use, and problems with machines.

## Results

The survey was sent to 352 individuals. A total of 46% (162/352) responded to parts of the survey, and 40% (140/352) completed it in its entirety.

Demographic information

**Figure 1:**
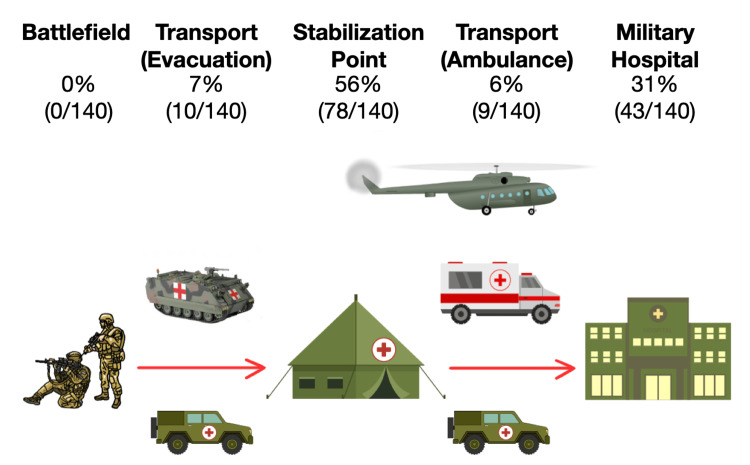
Patient flow and percentages of survey respondents that used POCUS in each setting (N = 140) The flow of soldiers injured in war. Soldiers typically first sustain an injury on the battlefield. They are evacuated to stabilization points, which have both emergency department and operating room capabilities. As needed, patients are transported to military and civilian hospitals for higher levels of inpatient care. Images were created by Kravchuk S, using Canva (Canva Inc., Perth, Australia) and edited in Keynote (Apple Inc.)

A total of 87% (122/140) of respondents were doctors, 4% (6/140) were medical assistants, 4% (6/140) were volunteers, 3% (4/140) were combat medics, and 1% (2/140) were instructors. A depiction of the components of longitudinal medical care in war is shown in Figure 1. A total of 56% (78/140) worked at stabilization points, 31% (43/140) worked in military hospitals, and 14% (19/140) worked in transport.

Ultrasound use

**Figure 2:**
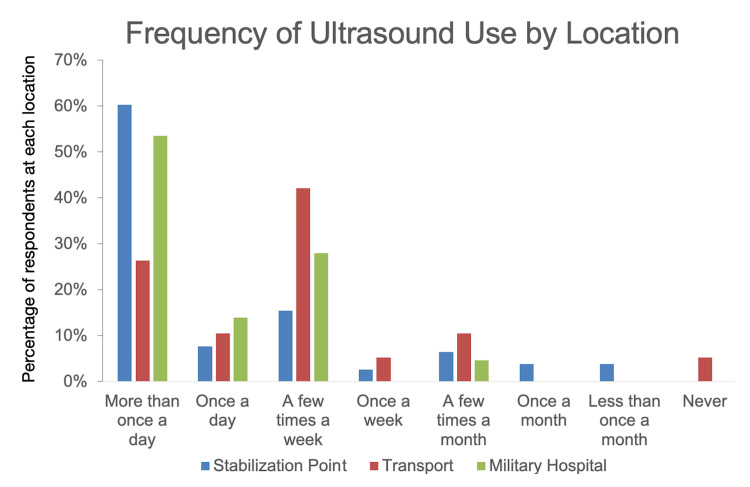
Frequency of use of POCUS broken down by location of care (N = 140) POCUS: point-of-care ultrasound Percentages of respondents at each location. A total of 56% (78/140) were at stabilization points, 13% (19/140) worked in transport (evacuation and ambulance), and 31% (43/130) were at military hospitals

A total of 69% (96/140) of respondents reported using a handheld ultrasound machine with a corded attachment to a screen. Also, 33% (46/140) used a traditional cart-based machine, and 27% (38/140) used a handheld machine that connected wirelessly to a screen. Note that the sum of responses is greater than 100%, as participants could report that they used more than one type of machine. A distribution of frequency of use broken down by area of work is shown in Figure 2.

**Figure 3:**
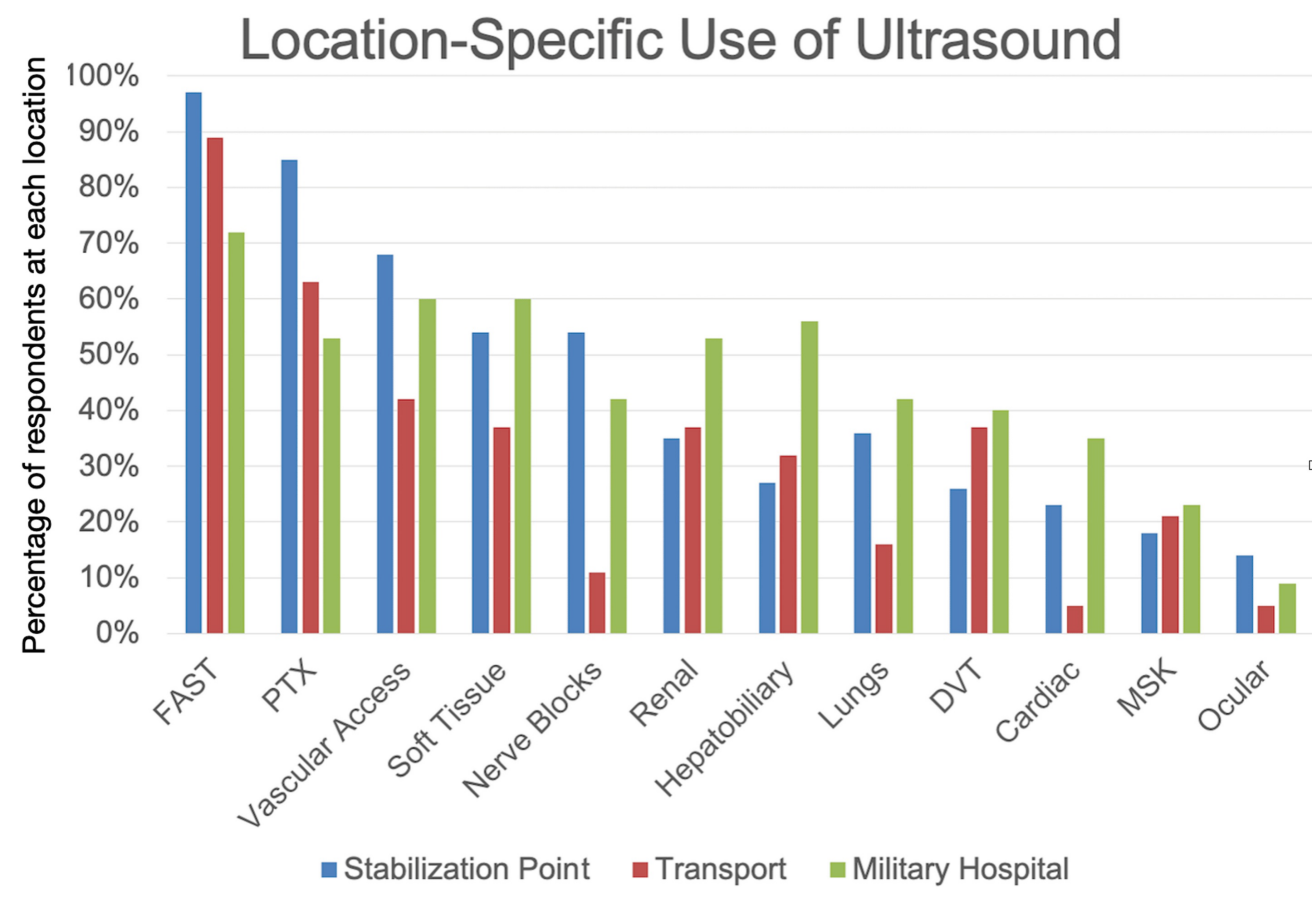
Use of various applications of ultrasound broken down by location of POCUS (N = 140) POCUS: point-of-care ultrasound Percentages represent the percentage of respondents at each location that perform each of the indicated applications. Note that percentages total greater than 100% as respondents could choose more than one application

POCUS was most used at stabilization points and at military hospitals (median and mode at both sites were “more than once a day”) and less so during transport (median and mode were “a few times a week”). Respondents were asked which POCUS applications they used. Of all respondents, the most commonly used POCUS applications were FAST (89% of respondents perform FAST exams), pneumothorax (72%), vascular access (62%), soft tissue (54%), and nerve blocks (44%). A breakdown of responses by location is shown in Figure 3.

Barriers to POCUS use

**Figure 4:**
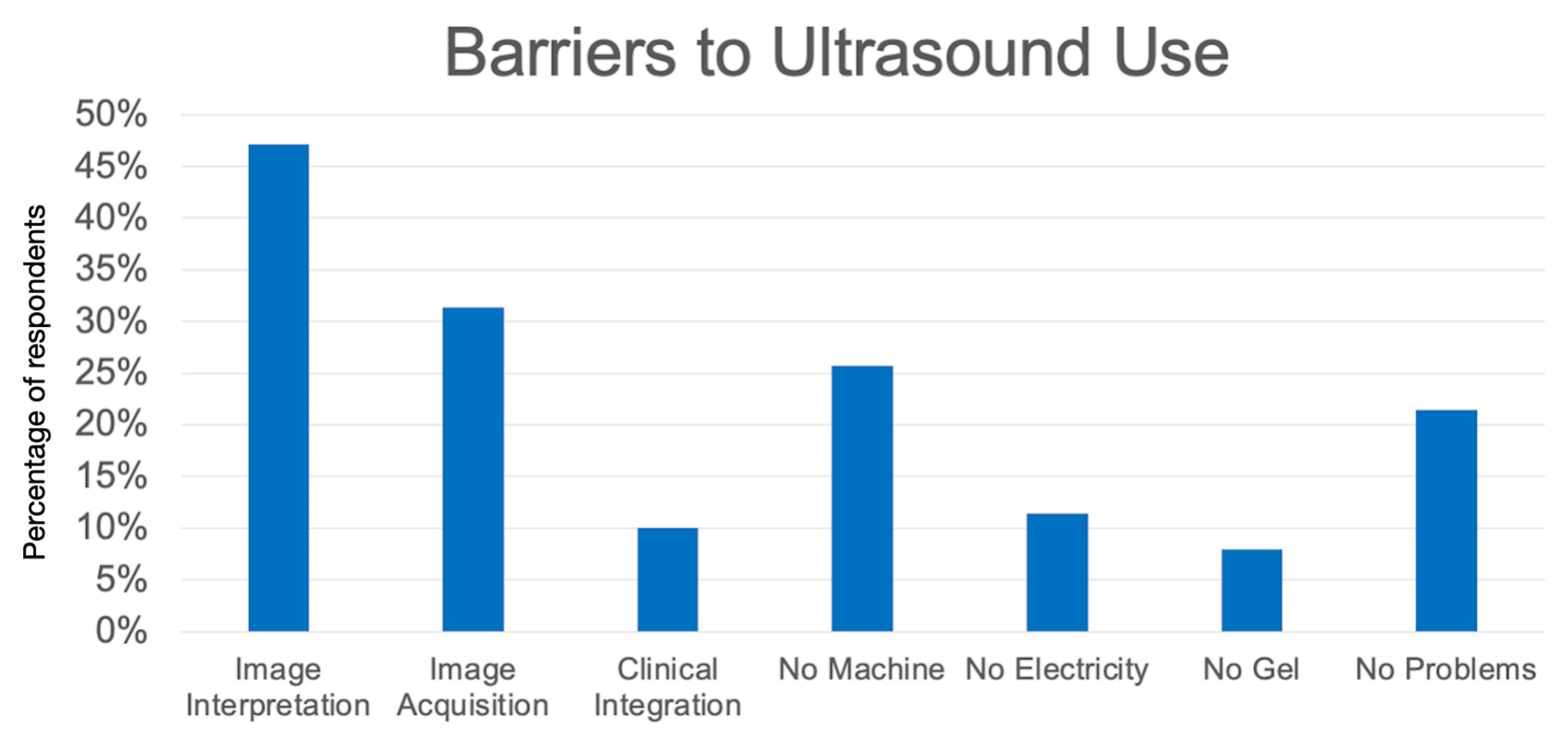
Educational and mechanical barriers to POCUS use (N = 140) POCUS: point-of-care ultrasound Note that participants could select multiple choices, and so percentages sum to greater than 100%

Respondents were asked about barriers to ultrasound use. Results are shown in Figure 4.

**Figure 5:**
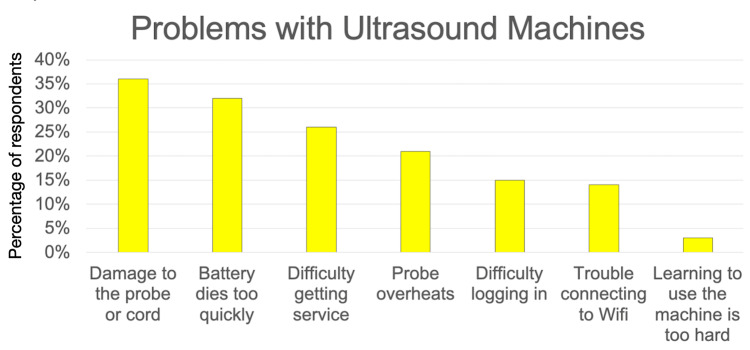
Issues with hardware, software, and connectivity (N = 140) Note that participants could select multiple choices, and so percentages sum to greater than 100%

Similarly, they were surveyed about what problems they encountered with ultrasound machines. Results are shown in Figure 5.

## Discussion

Over the past 4-5 years, POCUS has actively developed in Ukraine, especially in the military-medical environment. A significant contribution to this process has been made by international missions and trainings, which have been actively conducted since the beginning of the full-scale invasion [10]. Traditionally, formal ultrasound examinations can only be performed and interpreted by medical doctors who have completed special training, which can take months to complete. However, the concept of POCUS has changed this paradigm. As POCUS answers specific questions and is not meant to be a comprehensive assessment of every aspect of an organ, training paradigms are much simpler. Application-specific POCUS education can be done in a matter of days or even hours.

Similarly, the scope of trainees has expanded as well, with paramedics and nurses learning alongside doctors. There is precedent for nonphysician personnel with basic training to effectively use ultrasound in areas of armed conflicts [12-15]. Former applications that POCUS is useful for include the FAST exam, looking for abdominal fluid [7], ocular blast injuries [16], fractures [15], and thoracic injuries [8]. Finally, the miniaturization of ultrasound machines that has occurred over the past few years has dramatically changed the scope of where POCUS can be performed. POCUS is no longer confined to the walls of hospitals as handheld devices can be carried to remote stabilization points, on ambulances, and even to the battlefield.

Exactly which POCUS applications should be taught to medical personnel heading into a war zone is unknown. In an effort to better inform future POCUS training for medical personnel, in this study, we evaluated which applications were highest yield. Not surprisingly, assessing for free abdominal fluid and for pneumothorax with the eFAST exam were the most common uses. This makes sense as the bulk of injuries in war can be expected to be blunt and penetrating trauma. Ultrasound for vascular access was also commonly performed, which is also not surprising, as it is a staple of adequate resuscitation. We were surprised to see that soft tissue ultrasound was the next most common application; it seems from comments that its value is in identifying and guiding the removal of foreign bodies (especially shrapnel), which, without ultrasound, is sometimes done with magnets. And the fifth most commonly used application of POCUS was for regional anesthesia. This likely reflects the high volume of extremity injuries and the need for adequate pain control in conflict settings. Finally, we were surprised by how relatively infrequently cardiac ultrasound was performed. Assessing left and right ventricular function is one of the most common applications of POCUS in emergency departments in the US. Potentially, this discordance in our survey may be explained by the fact that heart failure and pulmonary embolism are not major clinical concerns in medical facilities providing care to military personnel who are typically able-bodied and healthy. De-emphasizing the education of cardiac POCUS in favor of higher-yield applications may be considered in future training sessions.

Location-wise, ultrasound was used with high frequency at stabilization points and in military hospitals. Use during evacuation and transport was less, with the highest number of respondents reporting using it “a few times a week.” This is probably because transport and evacuation times are typically brief and rapid and constrained by space. No respondents reported using ultrasound directly on the battlefield. In these circumstances, immediate life-saving interventions such as rapid hemorrhage control through self-aid or buddy aid are the primary clinical priority, and the use of ultrasound is often impractical. Accordingly, focusing future training and deployment efforts on personnel working at stabilization points or in military hospitals is likely to yield the greatest benefit.

Of the educational barriers to use, the most commonly cited was image interpretation. This challenge is likely not unique to Ukraine and may be expected in any setting where rapid, short-format POCUS training is provided to clinicians responding to armed conflict or humanitarian emergencies. Standards of comprehensive POCUS training in EM residencies exist and are far more extensive than what can be offered in a two-day course [17-19].

To what degree medical personnel are ready to competently and accurately use POCUS after a brief training is unknown. Errors may be made, but perhaps the benefits of releasing this technology, even with limited training, outweigh the risks. Focusing future training on repetitive interpretation of images may be useful in building skills and confidence. From a technical standpoint, 26% (36/140) of respondents reported a lack of access to a machine, suggesting that additional machines would be useful. These findings are similar to a previous study, in which primary barriers that doctors faced were a lack of machines and a lack of education [10]. Reassuringly, 21% (30/140) of respondents reported no barriers to performing POCUS.

For the wider use of ultrasound during the war in Ukraine, further institutionalization of POCUS is necessary. This includes the creation of educational programs on POCUS and their implementation in the training of civil and military medical personnel. It is necessary to develop standards and policies for the use of POCUS, which will regulate the use of ultrasound by medics of different specialties and levels. For example, the use of ultrasound could be mandated when providing assistance to the wounded at certain stages of evacuation. Until the institutional implementation of POCUS in Ukrainian medicine, advocacy for this method depends entirely on NGOs and individual initiative groups. Therefore, these initiatives need support to ensure the effective use of POCUS in wartime conditions.

There were numerous limitations to our study. Firstly, the survey was sent to the phones of medical personnel, sometimes many months after the course. It is possible that an individual may have lost their phone, not been in an area with cellular service or a secure wireless connection, or perished in combat. This may have led to a selection bias, though we do not speculate that this would have significantly affected our results in either a positive or a negative way. Second, participants from different training sessions were surveyed. The content and structure of these sessions were not standardized, and so it is possible that some participants received more or less training in certain uses of POCUS and that this may have affected their use and comfort with ultrasound. Third, resources of various stabilization points, transport, and hospitals may vary greatly, and potentially in ways that would affect the use of POCUS. The generalizability of the results may therefore be affected. Fourth, while having some clinical follow-up to ensure that POCUS was being used correctly would have been ideal, this was not feasible or practical for a number of reasons. To that end, we do not know to what degree, if any, images were being saved and stored for review, so we had no way of assessing if images were acquired or interpreted correctly. Additionally, as integration of clinical findings is dependent on the clinical resources and environment, it is hard to comment on whether the clinical decisions made based on POCUS findings were correct. And finally, participants were former students in POCUS training courses and may have felt compelled to answer questions positively. This may have skewed our data in a positive direction.

## Conclusions

After a brief POCUS training course, medical personnel in Ukraine used POCUS frequently to diagnose traumatic injuries, obtain vascular access, and perform regional anesthesia. POCUS use was greatest at stabilization points and in the hospital setting. Future efforts in POCUS education should increase focus on image interpretation. More ultrasound machines are needed.

## Appendices

Survey of Ukrainian Military Medics

Page 1

Record ID

__________________________________

Шановний учасник курсу з ультразвуку,

У рамках подальшого супроводу після курсу з ультразвукової діагностики, в якому ви брали участь, ми

проводимо анонімне опитування військових медиків щодо використання ультразвукової діагностики (УЗД) в

умовах воєнного часу.

Це опитування спрямоване на те, щоб охарактеризувати поширеність використання ультразвуку в рутинній

практиці військових медиків, з'ясувати пріоритетні напрямки (етапи надання допомоги) та способи його

використання зараз і в майбутньому. Дослідження також аналізує перешкоди для успішного використанняультразвуку серед військових медиків.

Усе це допоможе нам спрямувати додаткові тренінги з ультразвукової діагностики в майбутньому.

Участь в опитуванні є добровільною. Ви не зобов'язані брати участь у цьому дослідженні і можете в будь-якийчас припинити участь у дослідженні. Якщо ви вирішите не брати участь у цьому дослідженні або припините

участь пізніше, це жодним чином не вплине на вас.

Запрошуємо вас заповнити наступне опитування. Його заповнення займе приблизно 5 хвилин.

Відповіді на опитування є анонімними, і жодна ідентифікаційна інформація не буде передана будь-кому.

Заповнюючи це опитування, ви погоджуєтеся взяти участь у цьому дослідженні.

Дякуємо за вашу увагу та час!

Dear Prior Ultrasound Course participant,

As a follow-up to the ultrasound course in which you partook, we are conducting an anonymous survey of military

medics regarding the use of ultrasound diagnostics (USD) in wartime conditions.

This survey is designed to characterize the prevalence of the use of the ultrasound in the routine practice of military

medics, to find out the priority directions (stage of providing care) and ways of its use now and in the future. The

study also analyzes obstacles to the successful use of ultrasound among military medics.

All of this will help us to guide additional ultrasound trainings in the future.

Responding to this survey is voluntary. You do not have to participate in this study and may choose to leave the

study at any time. If you decide not to participate in this study or leave the study at a later time, you will not be

affected in any way.

We invite you to complete the following survey. It should take approximately 5 minutes to complete.

Survey responses are anonymous and no identifying information will be shared with anyone.

By completing this survey, you are agreeing to participate in this study.

Thank you for your consideration and time!

1) Військово-медична спеціальність? Medical military specialty? 

Санітар (aide)

Санітарний інструктор (instructor)

Бойовий медик (combat medic)

Фельдшер (medical assistant)

Медсестра (nurse)

Військовий лікар (military doctor)

Волонтер (medical volunteer)

Інше (other)

2) Рівень надання медичної допомоги, де ви переважно працюєте? What is the level of medical care where you mainly work? 

Допомога на полі бою (battlefield, first aid)

Евакуація (Evacuation)--Casevac

Евакуація (Evacuation)--Medevac

Стабілізаційний пункт (Stabilization point)

Швидка допомога (Ambulance)

Військовий госпіталь (Military hospital)

**3) Ваша спеціальність у цивільному житті? What is your specialty in civilian life?** 

Лікар (doctor)

Медсестра/Медбрат (nurse)

Медичний асистент (medical assistant)

Студент медичного навчального закладу (medical student)

Професія не пов'язана з медициною (profession not related to medicine)

**4) Як часто ви застосовуєте метод ультразвуку у своїй роботі? How often do you use ultrasound in your work?** 

Більше одного разу на день (More than once a day)

Один раз на день (Once a day)

Декілька разів на тиждень (A few times a week)

Один раз на тиждень (Once a week)

Декілька разів на місяць (A few times a month)

Один раз на місяць (Once a month)

Рідше одного разу на місяць (Less than once a month)

Ніколи (Never)

**5) На якому етапі ви використовуєте ультразвук? (оберіть всі, що застосовуються) What is the level of medical care where you mainly work? (check all that apply)** 

Допомога на полі бою (battlefield, first aid)

Евакуація (Evacuation)--Casevac

Евакуація (Evacuation)--Medevac

Стабілізаційний пункт (Stabilization point)

Швидка допомога (Ambulance)

Військовий госпіталь (Military hospital)

6) На вашу думку, де ультразвук є Допомога на полі бою найбільш корисним? (battlefield, first aid) In your opinion, where do you think ultrasound is the most useful?

Евакуація (Evacuation)--Casevac

Евакуація (Evacuation)--Medevac

Стабілізаційний пункт (Stabilization point)

Швидка допомога (Ambulance)

Військовий госпіталь (Military hospital)

7) Які застосування ультразвуку ви використовуєте? (оберіть всі, що застосовуються) Which ultrasound applications do you use? (check all that apply)

FAST-обстеження, пошук внутрішньої кровотечі (FAST examination, search for internal bleeding)

Виявлення пневмотораксу (Detection of pneumothorax)

Доступ до периферичних та центральних судин під ультразвуковим контролем (Access to peripheral and central vessels under ultrasound guidance)

Блокади нервів під ультразвуковим контролем (Nerve blocks accompanied by ultrasound)

Виявлення сторонніх тіл та ультразвукове обстеження м'яких тканин (Detection of foreign bodies and ultrasound of soft tissues)

Ультразвукове обстеження легень, діагностика пневмонії, набряку легенів та гідротораксу (Ultrasound of the lungs, diagnosis of pneumonia, pulmonary edema and hydrothorax)

Ультразвукове обстеження серця (Ultrasound of the heart)

Оцінка травм ока (Assessment of eye injury)

Ультразвукове обстеження печінки та жовчного міхура (Ultrasound of the liver and gallbladder)

Ультразвукове обстеження нирок (Ultrasound of the kidneys)

Оцінка наявності згустків крові в глибоких венах ніг (Evaluation of the presence of blood clots in the deep veins of the legs)

Оцінка опорно-рухового апарату, наприклад, переломи кісток, розриви сухожиль (Musculoskeletal assessment, e.g., bone fractures, tendon tears)

Інше (Other)

Якщо ви обрали "Інше", будь ласка, поясніть, які інші застосування ультразвуку ви використовуєте. If you said Other, then please explain.

8) Для кожного застосування, будь ласка, вкажіть, наскільки воно корисне для вас (1 означає зовсім не корисне, 7 - надзвичайно корисне). For each application, please indicate how useful it is to you (1 is not at all useful, 7 is extremely useful)

FAST-обстеження, пошук внутрішньої кровотечі (FAST examination, search for internal bleeding)

Виявлення пневмотораксу (Detection of pneumothorax)

Доступ до периферичних та центральних судин під ультразвуковим контролем (Access to peripheral and central vessels under ultrasound guidance)

Блокади нервів під ультразвуковим контролем (Nerve blocks accompanied by ultrasound)

Виявлення сторонніх тіл та ультразвукове обстеження м'яких тканин (Detection of foreign bodies and ultrasound of soft tissues)

Ультразвукове обстеження легень, діагностика пневмонії, набряку легенів та гідротораксу (Ultrasound of the lungs, diagnosis of pneumonia, pulmonary edema and hydrothorax)

Ультразвукове обстеження серця (Ultrasound of the heart)

Оцінка травм ока (Assessment of eye injury)

Ультразвукове обстеження печінки та жовчного міхура (Ultrasound of the liver and gallbladder)

Ультразвукове обстеження нирок (Ultrasound of the kidneys)

Оцінка наявності згустків крові в глибоких венах ніг (Evaluation of the presence of blood clots in the deep veins of the legs)

Оцінка опорно-рухового апарату, наприклад, переломи кісток, розриви сухожиль (Musculoskeletal assessment, e.g., bone fractures, tendon tears)

Інше (Other)

Якщо ви обрали "Інше", будь ласка, поясніть, які інші застосування ультразвуку ви використовуєте. If you said Other, then please explain.

9) Опишіть ситуацію в вашій практиці, коли ультразвук змінив ситуацію. Describe a situation in your practice where ultrasound changed the situation.

10) Що, на вашу думку, є перешкодою для успішного використання ультразвуку в вашій роботі? (Відзначте всі, що стосуються) What do you think is an obstacle to the successful use of ultrasound in your work? (check all that apply)

Відсутність навичок отримання зображень (Lack of skill in acquiring images)

Відсутність навичок інтерпретації зображень (Lack of skill in interpreting images)

Відсутність знань про те, що робити з результатами (Lack of knowledge of what to do with the findings)

Відсутність доступу до ультразвукового апарату (Lack of access to an ultrasound machine)

Відсутність гелю (Lack of gel)

Відсутність електрики (Lack of electricity)

Інше (Other)

Якщо ви обрали "Інше", будь ласка, поясніть, які інші застосування ультразвуку ви використовуєте. If you said Other, then please explain.

## References

[1] Moore CL, Copel JA (2011). Point-of-care ultrasonography. N Engl J Med.

[2] McNeil CR, McManus J, Mehta S (2009). The accuracy of portable ultrasonography to diagnose fractures in an austere environment. Prehosp Emerg Care.

[3] Henwood PC, Mackenzie DC, Rempell JS (2016). Intensive point-of-care ultrasound training with long-term follow-up in a cohort of Rwandan physicians. Trop Med Int Health.

[4] Henwood PC, Mackenzie DC, Rempell JS (2014). A practical guide to self-sustaining point-of-care ultrasound education programs in resource-limited settings. Ann Emerg Med.

[5] Poberezhets V (2022). Healthcare crisis in Ukraine-worrying consequences of the Russian-Ukrainian war. Croat Med J.

[6] Barten DG, Tin D, Granholm F, Rusnak D, van Osch F, Ciottone G (2023). Attacks on Ukrainian healthcare facilities during the first year of the full-scale Russian invasion of Ukraine. Confl Health.

[7] Miletić D, Fuckar Z, Mraović B, Dimec D, Mozetic V (1999). Ultrasonography in the evaluation of hemoperitoneum in war casualties. Mil Med.

[8] Brooks AJ, Price V, Simms M (2005). FAST on operational military deployment. Emerg Med J.

[9] Nelson BP, Melnick ER, Li J (2011). Portable ultrasound for remote environments, part I: feasibility of field deployment. J Emerg Med.

[10] Dieiev V, Dubrov S, Díaz-Gómez JL (2024). Point-of-care ultrasonography in Ukraine: a survey of anesthesiologists-intensivists participating in ultrasonography courses. Can J Anaesth.

[11] (2025). Butterfly Network and Razom expand global health partnership to bring additional 200 Butterfly iQ+ ultrasound systems to Ukraine. https://www.razomforukraine.org/butterfly-network-and-razom-expand-global-health-partnership-to-bring-additional-200butterfly-iq-ultrasound-systems-to-ukraine/.

[12] Hile DC, Morgan AR, Laselle BT, Bothwell JD (2012). Is point-of-care ultrasound accurate and useful in the hands of military medical technicians? A review of the literature. Mil Med.

[13] Monti JD, Perreault MD (2020). Impact of a 4-hour introductory eFAST training intervention among ultrasound-Naïve U.S. military medics. Mil Med.

[14] Salazar RF, Monti JD, Cronin AJ, Perreault MD, Naylor JF, Ahern BJ, Gendron BC (2021). Combat medic eFAST with novel and conventional portable ultrasound devices: a prospective, randomized, crossover trial. Med J (Ft Sam Houst Tex).

[15] Savell SC, Baldwin DS, Blessing A, Medelllin KL, Savell CB, Maddry JK (2021). Military use of point of care ultrasound (POCUS). J Spec Oper Med.

[16] Ritchie JV, Horne ST, Perry J, Gay D (2012). Ultrasound triage of ocular blast injury in the military emergency department. Mil Med.

[17] Lewiss RE, Tayal VS, Hoffmann B (2014). The core content of clinical ultrasonography fellowship training. Acad Emerg Med.

[18] (2023). Ultrasound guidelines: emergency, point-of-care, and clinical ultrasound guidelines in medicine. Ann Emerg Med.

[19] (2001). ACEP emergency ultrasound guidelines-2001. Ann Emerg Med.

